# Allometry of wing twist and camber in a flower chafer during free flight: How do wing deformations scale with body size?

**DOI:** 10.1098/rsos.171152

**Published:** 2017-10-18

**Authors:** Yonatan Meresman, Gal Ribak

**Affiliations:** 1 School of Zoology, Faculty of Life Sciences, Tel Aviv University, Tel Aviv 6997801, Israel; 2 The Steinhardt Museum of Natural History, Israel National Center for Biodiversity Studies, Tel Aviv, Israel

**Keywords:** coleoptera, dynamic bending, flapping flight, flexural stiffness, insect wings

## Abstract

Intraspecific variation in adult body mass can be particularly high in some insect species, mandating adjustment of the wing's structural properties to support the weight of the larger body mass in air. Insect wings elastically deform during flapping, dynamically changing the twist and camber of the relatively thin and flat aerofoil. We examined how wing deformations during free flight scale with body mass within a species of rose chafers (Coleoptera: *Protaetia cuprea*) in which individuals varied more than threefold in body mass (0.38–1.29 g). Beetles taking off voluntarily were filmed using three high-speed cameras and the instantaneous deformation of their wings during the flapping cycle was analysed. Flapping frequency decreased in larger beetles but, otherwise, flapping kinematics remained similar in both small and large beetles. Deflection of the wing chord-wise varied along the span, with average deflections at the proximal trailing edge higher by 0.2 and 0.197 wing lengths compared to the distal trailing edge in the downstroke and the upstroke, respectively. These deflections scaled with wing chord to the power of 1.0, implying a constant twist and camber despite the variations in wing and body size. This suggests that the allometric growth in wing size includes adjustment of the flexural stiffness of the wing structure to preserve wing twist and camber during flapping.

## 1. Introduction

Body size influences many aspects of living creatures [1] not the least of which is locomotion [2]. For flying animals, size imposes an upper limit for hovering [3] and muscle-powered flight [4], as well as dictating changes in wing skeletal and muscular structures [5]. Body size also affects flight speed [6], acceleration and manoeuvrability [7,8] and the energetic cost of aerial locomotion [9]. The relationship between body mass and these flight-related traits is often nonlinear and provides a statistical comparative tool with which to examine the function of different organisms within a wide range of size scales [10–16]. While scaling relationships should apply to both interspecific and intraspecific variation in body size, the scaling of flight-related traits within a single species can disclose the effect of physical size more accurately, by isolating it from variations associated with other ecological and phylogenetic factors.

Intraspecific variation in body size can be particularly high in insects. Some insects are sexually dimorphic in body size, and in many insects environmental effects and diet conditions during the larval phase can affect the somatic growth, resulting in high variation in the adult body size [17–20]. Such variation should be accompanied by an appropriate allometry of flight-related traits, including wing morphology [20–22], if the performance or efficiency of small and large individuals are to remain at the same level.

The flight wings of insects are thin and membranous structures, reinforced by thicker and stiffer veins. Unlike birds and bats, insects have no muscles within the wings, complicating direct control over the aerofoil shape. Nevertheless, insect wings elastically deform (bend and twist) during flapping as a result of the inertial and aerodynamic forces that emerge from the flapping motion [23–25]. Although the direct flight muscles of insects act on the base of the wings to fine-tune wingbeat kinematics, much of the change in wing twist and camber within each flapping cycle is accomplished by the mechanical properties of the elastic wing structure [26–28]. The adaptation of a wing's mechanical properties for ‘automatic’ adjustment of twist and camber during flapping flight poses an intriguing question: To what extent are such deformations tuned to optimize flight for insects of the same species but varying in body size? This question poses a complicated problem because alteration in wingbeat kinematics probably leads to alteration in inertial and aerodynamic forces, leading to alteration in wing deformation. However, altered wing deformation in itself leads to alteration of the flows over the wing, thus altering the aerodynamic force. Hence, wing deformation and the resulting aerodynamic forces are interlinked, resulting in a complex interaction that is difficult to predict [24].

Moreover, the elastic deformations that result from a given force depend on the flexural stiffness of the wing [23]. Flexural stiffness (or flexural rigidity, ‘EI’; see equation (1.1) below) is a term representing an object's (here a wing's) resistance to bending [29,30]. It is used in engineering disciplines to predict deflections in simple beams made of uniform materials and cross sections. However, the structure and material properties of insect wings are too complex to allow simple mathematical predictions of elastic shape deformations during flapping [31,32]. It is well accepted that some ability of the wing to deform during flapping is needed in order to allow efficient lift production within a single flapping cycle, by means of a favourable wing camber and lengthwise wing twist [28,33,34]. However, there is still controversy over whether the deformations improve [34–43] or impair [44,45] aerodynamic output compared to a rigid wing, and to what extent. Consequently, invaluable insight could be gained by measuring wing deformation during flapping flight in free-flying insects and linking these deformations to the wing morphology and flapping kinematics.

While insect wings are highly diverse in shape and size, they share several preserved characteristics: they tend to have thicker and more numerous veins towards the leading edge of the wings and towards the wing base. These veins increase the flexural stiffness of these regions [23] resulting in lengthwise and chord-wise gradients in flexural stiffness. These gradients result in the typical patterns and reversals of wing twist (lengthwise) and camber (bending chord-wise).

Combes & Daniel [23] measured the static bending of wings removed from various insects, and used a simple cantilever beam model to calculate the flexural stiffness of the wings. They demonstrated that the flexural stiffness scales with the cubic power of wing span and the square power of wing chord for span-wise and chord-wise deflections, respectively. This indicates that larger wings are stiffer and that when the same force is applied to wings varying in size, the deformation should scale with span to the power of 0 and chord to the power of 1. However, the aerodynamic force on a larger wing should be higher due to higher wing loading (weight/wing area), which roughly scales with length to the power of 1 [46].

For a cantilever beam of length *l* loaded with equally distributed load (*ω*) the deflection (*β*) at the free end is [30]:

1.1 $$\beta =\frac{\omega l^{4}}{8\text{EI}}.$$

The load per metre (*ω*) is proportional to the square power of length (wing-loading × length) This suggests that deformation along the chord can increase with the chord raised to the power of approximately 4, implying that aerodynamic force can result in larger camber (deflection/chord) in larger insects.

This prediction, derived from cantilever beam theory, is a gross oversimplification for elastic insect wings. It assumes isometric growth of length and beam cross section (width and thickness). The aerodynamic force acting on the wings during flapping is not locally or uniformly distributed over the wing area and it varies with time within the flapping cycle. Furthermore, deflection of a simple beam during dynamic loading can double compared to static loading by the same force [47]. In addition, inertial forces that are generated during flapping (mainly during wing stroke reversals) play a greater role in wing deformation than aerodynamic forces [48]. These inertial forces depend on the thickness of the wing and distance from the wing hinge, and both inertial and aerodynamic forces depend on the flapping kinematics [49], which also typically change with body size (e.g. wingbeat frequency [50,51]). Consequently, although scaling arguments may hint at proportionally larger wing deformations along the wing chord in larger insects, empirical measurements on free-flying insects are needed in order to evaluate the actual relationship between wing size and deformations during flight. When variability of body size is high within a population, this provides a natural experiment by which to determine such scaling relationships.

**Figure 1.**
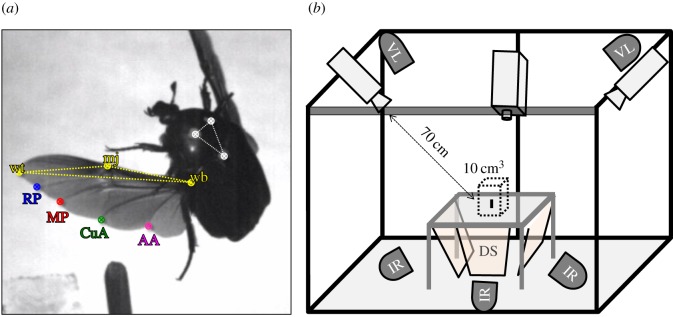
Methodology. (*a*) A frame from a high-speed film showing the free-flying *Protaetia cuprea* and the digitized landmarks. White dots on the thorax were used to extract body orientation. Colour-coded dots denote wing landmarks used to extract flapping kinematics and wing deflection at the trailing edge: wb, wing base; wt, wing tip; mj, marginal joint; AA, analis-anterior vein; CuA, cubitus-anterior vein; RP, radius-posterior vein; MP, media-posterior vein (*sensu* [52]). Triangles illustrate the two-dimensional planes that are defined using three points (appendix B). (*b*) Schematic illustration (not to scale) of the experimental set-up. Three high-speed cameras were placed above a calibrated mutual field of view from which the beetles took off. Infrared (IR) floodlights behind diffusive screens (DS) from below and two visible floodlights (VL) from above provided illumination for the cameras and beetles, respectively.

Here, we examined how wing deformation during flapping changes among individual rose chafers (figure 1*a*) differing almost threefold in body mass. *Protaetia cuprea* Fabricius (1775) (Scarabaeidae: Cetoniinae) are considered to be accomplished flyers within the Scarab family. They are fruit and pollen feeders that fly during the day, requiring accurate manoeuvring flight in order to land precisely on top of a flower or fruit. To the best of our knowledge, the structural adaptations of rose-chafer wings to manoeuvring flight have not been studied to date. We hypothesized that the wings of larger beetles would deform more due to the nonlinear scaling relationship between wing length and elastic deflection. This would lead to changes in twist and camber of the wings during flapping. Alternatively, if wing twist and camber are important flight-related traits, then deflection of the trailing edge during flapping should increase in proportion to the wing chord and larger wings should stiffen to preserve twist and camber. To test which of these hypotheses is correct, we used high-speed cameras to measure the wing deformation of the beetles during free flight.

## 2. Material and methods

### 2.1. Insects

Seventeen adult rose chafers (10 females and 7 males), varying in body size (minimum and maximum body mass: 0.38 g and 1.29 g, respectively), were selected to represent the range of body sizes in the natural population [53]. As different functional demands of reproduction, as well as sexual selection may lead to sexual dimorphism in body size within a given species, we first compared body mass and wing morphology between the sexes. *Protaetia cuprea* were found to be sexually monomorphic in both body mass and wing morphology (independent sample *t*-test, d.f. = 15; body mass: *t* = −0.308, *p* = 0.762; wing length: *t* = 0.384, *p* = 0.706; wing area: *t* = 0.176, *p* = 0.863; *R*_2_: *t* = 0.504, *p* = 0.622; wing loading: 0.686, *p* = 0.503). Similarly, we found no statistical evidence for difference in wing deflection between the two sexes (see Discussion). Therefore, we pooled the data from males and females together. The beetles were either collected during 2014–2016 from field sites in central Israel or taken from a population cultivated in the laboratory. The smallest and largest beetles in the study (body mass) were collected in the field.

First, the body mass of each beetle was measured to the nearest 0.1 mg and three white dots were painted on the dorsal side of the thorax using acrylic paint: two dots on either side of the pronotum and a third one on the scutellum (figure 1*a*). The mass of the paint was negligible (less than 0.1 mg, i.e. less than 0.2% of body mass) and we did not observe behavioural side effects associated with marking the beetles. The three dots allowed tracking the orientation of the body in the high-speed films. The wings were not marked to avoid obstruction of normal flapping kinematics and wing flexibility. Instead, we used seven natural morphological landmarks, comprising wing edge–vein junctions and inter-vein joints: the radius-posterior (RP), media-posterior (MP), cubitus-anterior (CuA) and the analis-anterior (AA) veins, as well as the marginal joint that enables the folding of the wing while at rest [52] (figure 1*a*). Wing length was measured from the wing base to the wing tip (‘wb’ and ‘wt’, respectively, in figure 1). Wing area, aspect ratio (wing span divided by the mean wing chord) and the second moment of wing area were measured and calculated based on scaled images of scanned wings (1200 dpi, HP OfficeJet 6700). Wings of two of the filmed beetles were not available for the morphological analysis.

### 2.2. Filming system and experiment protocol

Each beetle was placed on a vertical, 4 cm long, wooden rod (diameter = 3 mm) and allowed to take off voluntarily. Take-off was recorded 10 times or until the beetle was reluctant to fly, whichever occurred first. Each film showed the take-off and 15–45 flapping cycles that followed. We were able to extract useful data (see criteria below) from only 14 of the 17 beetles. The height above ground from which the beetles took off was slightly less than twofold the mean wing length (wing length: 2.19 cm, s.e. = ±0.05, *n* = 14) and we discarded at least five of the first flapping cycles to ensure that transient and ground effects [54,55] were negligible.

Take-offs were captured using three synchronized and spatially calibrated [56] high-speed cameras (Fastcam SA3_120 K, Photron inc.) fitted with 50 mm lenses and set to film at 3000 frames s^−1^ (shutter speed: 66.7 µs, resolution: 768 × 768 pixels)_._ The average wingbeat frequency of *P. cuprea* was 109 Hz (s.e. = ±2 Hz, *n* = 14), implying approximately 28 frames of captured high-speed video per flapping cycle. The cameras were positioned at a distance of approximately 70 cm from the beetle with an elevated viewpoint of approximately 45° (figure 1*b*). Three infrared (IR, wavelength 850 nm) LED floodlights (ELIMEC Ltd, angle 25°) generated diffused backlight for each of the cameras and two 500 W halogen floodlights from above provided visible light and illuminated the beetle directly (figure 1*b*). The sensitivity of *P. cuprea* to near IR is unknown. However, most insects are insensitive to IR light [57] and our beetles took off and flew upwards towards the visible lights, seemingly oblivious to the IR floodlights from below. The three calibrated cameras allowed measurement of the three-dimensional (3D) position at a minimal error of ±0.13 mm within a volume of 1000 cm^3^ mutually viewed by all three cameras.

### 2.3. Pre-analysis criteria

We selected one video for each beetle, fulfilling three pre-established criteria: (i) the beetle took off vertically without manoeuvring sideways, (ii) the vertical path occurred within the calibrated volume seen by all three cameras, and (iii) the beetle performed at least three (bilaterally) symmetrical flapping cycles following at least five flapping cycles after leaving the rod. These criteria resulted in 14 films (out of *n* = 17 beetles) that were manually digitized for the position of 10 landmarks (figure 1*a*) in each film frame of three consecutive flapping cycles. The digitized data were used to extract 3D coordinates for each landmark using DLTdv5 [56]. As straight-flight trajectories were selected (criterion (i)) and the flapping of the left and right wings was symmetrical (criterion (iii)), we only digitized landmarks from the left wing.

### 2.4. Data analysis

The instantaneous flight speed and mean acceleration of the beetles were found from the first and second time derivatives (respectively) of the instantaneous positions of the scutellum (lower white point in figure 1*a*). The time series of position data were fitted with second-order polynomial equations using standard Matlab (The Mathworks Inc.) functions to obtain the time derivatives in the three spatial axes. The mean *R*^2^ (±s.e., *n* = 14) values for the polynomial curve fitting functions on the *X*, *Y* and *Z* axes were 0.981 (±0.007), 0.980 (±0.008) and 0.997 (±0.002), respectively.

Using the three points on the thorax, we found the three major body axes in 3D space for each video frame. These axes were used to transform all instantaneous landmark positions on the wing to a body frame of reference (appendix A). We then used the three leading edge landmarks (wb, mj and wt in figure 1*a*) to define an instantaneous plane in 3D space that represents the (relatively) rigid surface of the wing. Next, we calculated for the four remaining landmarks (RP, MP, CuA and AA on the trailing edge; figure 1*a*) the perpendicular distance out of the defined plane (appendix B). These distances represent the maximum chord-wise deflections at these positions along the wing, relative to the rigid leading edge. The angle between mj and wt at wb is small, limiting the accuracy of finding the plane in 3D. We evaluated the associated error by recalculating deflections after expanding the area of the rigid leading edge by replacing point wt with RP. As expected from inclusion of less rigid wing area, the remeasured deflections relative to the leading edge area were slightly smaller, but the deformation pattern remained similar (electronic supplementary material, S1).

Another potential source of error for defining the plane of a rigid wing is span-wise wing deflection along the leading edge. We evaluated this error by measuring the deflection of three points between mj and wt from three of the filmed beetles, and found that the maximal measured span-wise deflection could account to an error of up to 3.4° in our measurement of the plane of the rigid wing (electronic supplementary material, S2). The chord-wise deflection angles are much larger, typically 20–40° (in the distal and proximal wing sections, respectively; see Results). The span-wise deflection of the wing tip is towards the ventral side during the mid-downstroke, implying that the small error from span-wise deflection results in a slight underestimation of the chord-wise wing deflection relative to the plane of the rigid wing. Our measurements and analysis of the chord-wise wing deflections always pertain to the mid-downstroke and mid-upstroke where the error due to span-wise deflection and wing rotational effects (see below) are small.

The film sequences analysed were three flapping cycles long (approx. 30 ms). To correct for changes in wingbeat frequency, we divided the serial numbers of each film frame by the number of frames within a flapping cycle, giving a normalized time scale of flapping cycles in the range 0–3.0. To obtain the allometric relationships between wing deformation and body size, we ignored the time-varying deformations during stroke reversals and selected the relatively fixed (see Results) deformation of the wing during the mid-downstroke and mid-upstroke. Data from the three consecutive flapping cycles for each landmark were averaged.

Our primary objective was to evaluate wing deformation in beetles differing in body size. However, our wing deformation data were measured from free-flying insects so that wing deformations were affected not only by size but also by the aerodynamic force required from the wings and intrinsic inertial forces. Ideally, wing deformation of all the beetles would be measured during hovering where net aerodynamic forces would exactly equal body weight. However, the free flying beetles never engaged in perfect hovering. As the beetles were filmed taking off, the aerodynamic forces generated by their wings should have exceeded body weight, allowing an upwards acceleration. The vertical acceleration of our beetles, *a*_v_, varied between −2.484 and 2.467 m s^−2^. All beetles flew upwards, thus positive (most beetles) and negative (one beetle) acceleration vectors imply acceleration and deceleration of the upwards speed, respectively. As *a*_v_ varied among the beetles, we examined the relationship of wing deformation once with the mass of the beetles and a second time with the mass multiplied by gravity + vertical acceleration *M*(*g* + *a*_v_). The latter served as a proxy for the time-averaged vertical force (*F*_V_) generated by the beetles. The vertical force only accounts for the net upthrust that propels the body upwards. The average aerodynamic forces on the flapping wing should be higher due to air resistance on the wings and the presence of an additional horizontal thrust component. Furthermore, wing deflections and aerodynamic forces both rely on flapping kinematics. We thus evaluated how much of the observed wing deformation could be explained by variation in wing size and wingbeat kinematics. To do so, we extracted wingbeat kinematics and used a simplification of the quasi-steady blade-element model [58] for the aerodynamic force generated by the wing during hovering flapping flight as in [59–61]:

2.1 $$\text{Aerodynamic force}\propto \frac{\rho \Omega^{2}f^{2}R^{2}S{\hat{r}}_{2}^{2}}{8}\sqrt{C_{D}^{2}+C_{L}^{2}},$$

where *ρ* is the air density, Ω is the flapping amplitude (in radians), *f* is the flapping frequency (in Hz), *R* is the wing length (in m), S is the area of the hindwings and ${\hat{r}}_{2}$ is the non-dimensional radius of the second moment of wing area. Here, *C*_D_ and *C*_L_ are the drag and lift force coefficients of the wing, respectively. The estimation of aerodynamic force (henceforth, EAF) shown above accounts for the mean (over the flapping cycle) aerodynamic force that is due to wing translation. It is the dominant force during the mid-half stroke, where the wing deformations were measured. Instantaneous rotational and inertial contributions to aerodynamic force, which are important in insect flight, have been shown theoretically as well as empirically to be negligible at that phase of the wing stroke [49,62]. The force estimate (equation (2.1)) is calculated relative to the stroke-plane angle, which will be shown below to be invariant with beetle size (see Results). The force coefficients (*C*_L_ and *C*_D_) are a trigonometric function of the angle of attack of the wing. Although wing flapping angles were invariant with body mass (see Results below), the beetles were flying upwards so that their climb speed could have had a direct effect on the aerodynamic angle of attack of the wing. We, therefore, estimated the inflow ratio (air velocity perpendicular to the stroke plane divided by air velocity in the stroke plane) of each beetle. This was done by finding the induced velocity from momentum theory and adding the climb speed to it [63]. The induced velocity (*v_i_*) of an actuator disc during a climb is [63]:

2.2 $$v_{i}=-\frac{V_{c}}{2}+\sqrt{{\left(\frac{V_{c}}{2}\right)}^{2}+\frac{T}{2\rho A}}.$$

where *V*_c_ is the climbing speed, *T* is the thrust generated by the disc (approximately body weight in low flight speeds) and *A* is the disc area (Ω*R*^2^).

The inflow ratio is

2.3 $$\lambda =\frac{V_{c}+v_{i}}{\Omega Rf}.$$

Equations (2.2) and (2.3) are for a horizontal actuator disc during a vertical climb. The stroke-plane angles of our beetles were indeed approximately horizontal (average 2° ± 8.4° from the horizontal). Nevertheless, to minimize errors associated with variation in the stroke-plane angle and climb speed, we always used the air velocity perpendicular to the stroke plane. Thus, for *V*_c_ in equations (2.2) and (2.3) we used the component of the climb speed that is perpendicular to the stroke plane (i.e. $V_{c}=V_{z}\cos\gamma$), where *V*_z_ is the vertical speed of the beetle and *γ* is the stroke-plane angle relative to the horizontal plane. The correction was very small; for the beetle with the most tilted stroke plane *V*_c_ was smaller than *V*_z_ by less than 4%.

The aerodynamic angle of attack relevant to the blade-element analysis is thus

2.4 $$\alpha =\;\theta -\tan^{-1}\lambda .$$

The resulting angle of attack was at the most 1.7° smaller than the angle of attack for true hovering (mean difference ± s.e. = 0.85° ± 0.11°, *n* = 14).

Owing to lack of available data on aerodynamic properties of flapping beetle wings, we generalized data for insect wings in general from [64] and used the trigonometric relationships

2.5*a* $$C_{L}=3\sin\alpha \cos\alpha$$

and

2.5b $$C_{D}=3.35\;\text{si}{\text{n}}^{2}\alpha +0.1,$$

to find the force coefficients to substitute in equation (2.1) (see electronic supplementary material, S3).

Finally, we evaluated how flapping inertial forces should vary with beetle size. This was done by measuring the distribution of mass along the left wing in five beetles. The wings were removed and cut along the chord into four segments that were measured for weight and length separately. The mass of wing sections was used to estimate the non-dimensional radius of the mass moment of inertia of the wing, and an estimate of inertial torque for each of the beetles in the study (see electronic supplementary material, S4).

### 2.5. Statistics

One-tailed paired *t*-tests were used to compare the deformations of the landmarks between the upstroke and the downstroke. We used the one-tailed test because greater flexion towards the ventral side compared to the dorsal side of the wing was expected [65,66]. One-way repeated-measures ANOVA (RMANOVA) was used to compare the magnitude of the deformations between the four landmarks on the trailing edge within each beetle. The test was performed once for the deformation during mid-downstroke and a second time during mid-upstroke. As sphericity could not be assumed, we used the Greenhouse--Geisser corrected value for both tests. Post hoc tests were performed by pair comparison using two-tailed paired *t*-tests with Bonferroni correction for multiple comparisons. Unless stated otherwise, all averages are reported below ± 1 s.e. and the sample size is *n* = 14.

Flapping kinematics were expected to change with body mass. We used linear regression on log-transformed data to evaluate the allometric relationships between body mass and wingbeat frequency and body mass and wingspan. The relationship between body mass and flapping amplitude was evaluated using only Pearson correlation because it did not show linearity.

Allometric relationships between wing deflection and forces were evaluated using linear regression on log-transformed data and the relationships were validated using 1000 bootstrap samples to test for estimation errors due to the relatively small range of body mass variation within our beetles.

## 3. Results

### 3.1. Pattern of wing deformation

**Figure 2.**
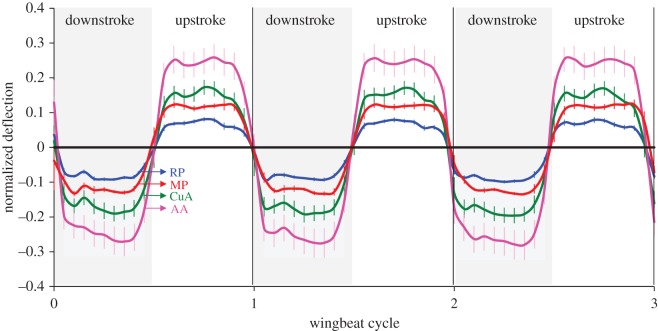
Wing deflections. Mean (error bars are ± 1 s.e.) deflections at the trailing edge during free flight in three consecutive flapping cycles (*n* = 14 beetles). Deflections are normalized by wing length and time is normalized by the flapping cycle of each beetle. Positive and negative values signify deflection towards the anatomical dorsal and ventral sides of the wing, respectively. Black denotes the relative null deflection of landmarks on the leading edge; magenta, green, red and blue denote deflections of landmarks on the trailing edge (AA, CuA, MP and RP, respectively; figure 1*a*).

The time-varying wing deformations of *P. cuprea* had a clear cyclic pattern that concurred with the flapping kinematics (figure 2; electronic supplementary material, S1). The landmarks on the trailing edge deflected out of plane towards the pressure side of the wing, so that during the downstroke and the upstroke the landmarks were deflected towards the anatomical ventral and dorsal sides of the wing, respectively. The magnitudes of the deflections during the downstroke were slightly, yet significantly, greater than during the upstroke (table 1). In addition, the magnitude of deflection differed between the various landmarks within both the downstroke and upstroke (One-way RMANOVA, downstroke: *F*_1.158, 15.051_ = 365.405, *p* < 0.001; upstroke: *F*_1.420, 18.463_ = 345.302, *p* < 0.001; figure 2). Post hoc paired comparisons revealed that proximal points on the trailing edge deflected significantly more than distal landmarks (electronic supplementary material, S5).

**Table 1.** Student's paired *t*-test results for the difference in wing deflection between mid-downstroke and mid-upstroke at the different landmarks*.*

	difference (mm) (downstroke)– (upstroke)			
landmark	mean	s.e.	*t*_(d.f.__=__13)_	*p*-value
RP	0.344	0.112	3.606	0.002
MP	0.572	0.145	3.936	0.001
CuA	0.503	0.149	3.378	0.002
AA	0.418	0.112	3.735	0.001

### 3.2. Flapping kinematics

**Figure 3.**
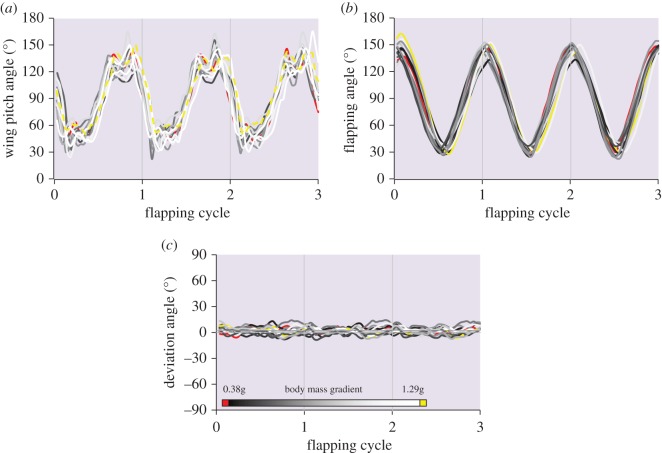
Flapping kinematics. Time-varying wing pitch (*a*), flapping (*b*) and deviation (*c*) angles of the 14 beetles used in the study during three consecutive flapping cycles. Greyscale colour code denotes ranked body mass from lowest (dark) to highest (light). The smallest and largest beetles are denoted by red and yellow, respectively.

The stroke-plane angle (relative to the longitudinal body axis) was 29.82° ± 0.65° and was invariant with size (Pearson correlation, *r* = 0.133, *p* = 0.649). The wing pitch (the angle between the stroke plane and the chord at point RP) was also consistent among beetles, with no apparent association to beetle body mass (figure 3*a*). Similarly, the instantaneous flapping and deviation angles (figure 3*b,c*) did not vary with body size.

**Figure 4.**
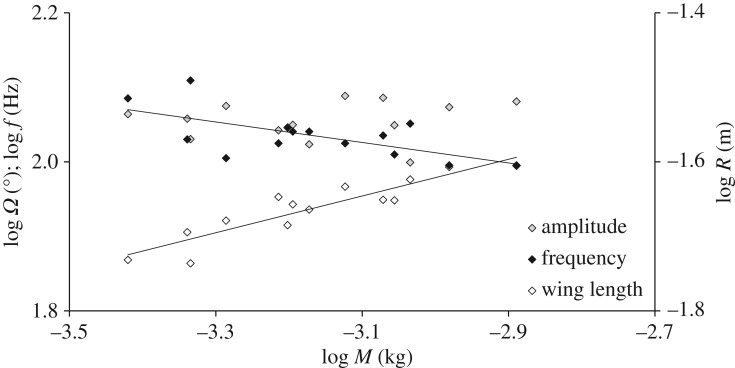
Logarithmic relationships of morphological and kinematic parameters with body mass (*M*). Black-, grey- and white-filled rhombi mark the frequency (*f*), amplitude (*Ω*) and wing length (*R*) data, respectively. Linear regression lines are displayed only in frequency and wing length data, where the model was statistically significant (*p* < 0.05).

Figure 4 presents the relationship of wingbeat frequency, flapping amplitude and wing length with body mass. Wingbeat frequency (*f,* minimum–maximum range: 99–129 Hz) decreased with increasing body mass (linear regression on log-transformed data, *R*^2^ = 0.414, *p* *=* 0.013, *n* = 14; figure 4) according to

3.1 $$f=\;39.811M^{-0.137},$$

where *f* is in Hz and body mass (*M*) is in kg. The flapping amplitude (mean ± s.e.) was 113.6° ± 1.77° (*n* = 14) and did not correlate significantly with body mass (*r* = 0.144, *p* = 0.624, *n* = 14; on log-transformed data; figure 4).

The relationship between wing length (*R*) and body mass (linear regression on log-transformed data, *R*^2^ = 0.837, *p* < 0.001, *n* = 14; figure 4) was found to be

3.2 $$R=\;0.131M^{0.246},$$

where *R* is in metres.

Our estimation of the mean aerodynamic force (EAF) was slightly better at predicting deflection compared to both body mass and vertical force (*F*_V_) (electronic supplementary material, S6). However, EAF had a significant linear relationship with both body mass and *F*_V_ (linear regressions, *p* < 0.001 in both cases; *R*^2^ = 0.773 and 0.797, respectively; electronic supplementary material, S7). The inter-correlation between the three force estimates and the improved correlation between EAF and the deflection encouraged us to use EAF as the proxy for the aerodynamic force on the wing. Estimates of wing inertial torque were linearly related to the *F_V_* (linear regression on log-transformed data, *p* < 0.001, *R*^2^ = 0.774) and scaled with body mass^1.15^, with the exponent not significantly different from 1.0 (electronic supplementary material, S4b(i)).

### 3.3. Scaling of wing deformations

**Figure 5.**
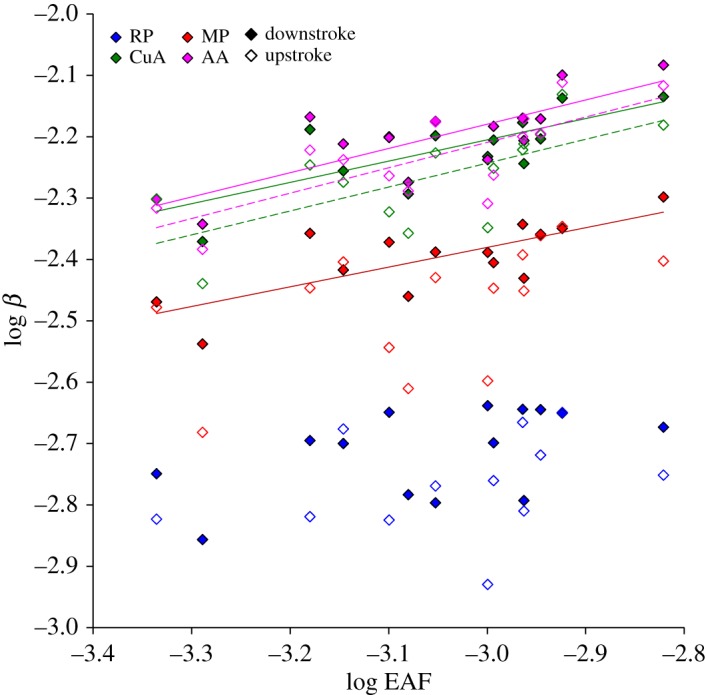
Logarithmic relationship between wing deflection (*β*) and the estimation of aerodynamic force (EAF). Filled and open symbols denote deflections during mid-downstroke and mid-upstroke, respectively. Colours and landmark names are as in figure 1*a*. Statistically significant linear regressions (on log-transformed data) are denoted by a trend line; see table 2 for elaborated statistics.

The linear regression between log-transformed landmark deflection and EAF was significant in MP, CuA and AA during the downstroke. The same held true for CuA and AA during the upstroke (linear regression; table 2 and figure 5). The deflection (*β*) at a specific landmark as a function of aerodynamic force (*F*_EAF_) could be described as a power function:

3.3 $$\beta =\;b{\left(F_{\mathrm{EAF}}\right)}^{a},$$

by substituting the parameters *a* and *b* with the relevant values given in table 2. Electronic supplementary material, S6 provides these coefficients using *F*_V_ and body mass instead of *F*_EAF_.

**Table 2.** Allometric coefficients (with the 95% CI in brackets) and linearity tests (on log-transformed data; figure 5) for the deflection of the different landmarks as a function of an estimate of aerodynamic force (EAF). Statistical significance (*p* < 0.05) is denoted by italic font. All results are based on 1000 bootstrap samples.

		coefficients			
stroke	landmark	exponent (a)	constant (b)	*R*^2^	*p*-value for linearity test
down	RP	0.254 (0.036, 0.485)	0.012 (0.002, 0.058)	0.263	0.061
	MP	0.321 (0.116, 0.492)	0.038 (0.009, 0.125)	0.558	*0.002*
	CuA	0.347 (0.147, 0.504)	0.069 (0.017, 0.200)	0.589	*0.001*
	AA	0.395 (0.180, 0.534)	0.101 (0.023, 0.265)	0.640	*0.001*
up	RP	0.414 (0.027, 0.926)	0.029 (0.002, 0.978)	0.217	0.093
	MP	0.362 (0.057, 0.802)	0.043 (0.005, 0.937)	0.266	0.059
	CuA	0.390 (0.177, 0.696)	0.084 (0.018, 0.724)	0.467	*0.007*
	AA	0.414 (0.235, 0.601)	0.107 (0.030, 0.386)	0.583	*0.001*

## 4. Discussion

Morphological parameters do not change evenly with changes in body size. Consequently, large individuals are seldom an isometric magnification of small ones, and the deviation from isometric growth can point to important structural and dimensional constraints on function. Elastic deflections along the wing chord of insects are believed to be important functional adaptations that convert the relatively flat and thin membranous wings of insects into aerofoils that can produce higher amounts of lift on both the downstroke and the upstroke [33]. However, the efficiency of elastic wings compared to rigid ones is still debated, partially because it is hard to generalize an ‘elastic insect wing’ out of the large variability in insect wing shapes, sizes and flapping kinematics. Here, by focusing on intraspecific variation in naturally occurring wing deflections during free flight, we isolated the effect of insect size on wing deflection from interspecific variability associated with phylogeny, flight ecology and wing shape.

We found a distinct pattern of deflection for points on the trailing edge that repeated itself during the flapping cycles. Similar to previous reports in other insects [67,68], the trailing edge deflected below the plane defined by the leading edge during the downstroke. Consequently, the wing had a positive camber as it translated towards the ventral stroke-reversal point. During the upstroke, the trailing edge deflected in the opposite direction relative to the plane of a rigid wing. However, because the wing now moved with the opposite side facing the oncoming flow, the result was again a positive camber. During stroke reversals, the deflections changed side very rapidly (within 1 ms). Thus, the wing was cambered almost throughout the translation phase of the flapping cycle, with the deflections remaining fairly constant during the wing translation phase. Hence, the deflections during mid-strokes are representative of the maximal chord-wise wing deformation observed during the translation phase. The mid-stroke is also associated with a maximum peak in the instantaneous aerodynamic forces that result from wing translation (*sensu* [49,69]). The deflection of the wings span-wise at these mid-strokes (judging by the deflection of wt compared to proximal points on the leading edge; electronic supplementary material, S2) was one order of magnitude smaller than the proximal chord-wise deflections reported above.

Despite the positive camber on both the downstroke and upstroke, we found small (7–21%) but significant differences in the magnitude of deflection of the same landmarks on the trailing edge between the two half-strokes. The larger deflection during the downstroke can result from a pre-existing curvature of the relaxed wing (YM, personal observation). However, it may also represent structural properties for anisotropic deflection of the wing planform due to forces applied at the dorsal or ventral sides of the wing. Such differences in the magnitude of deflections due to the same force being applied dorsally or ventrally have been reported before from direct deflection measurements on isolated insect wings [70,71]. These differences are believed to reflect an adaptation of the wing structure to higher loading during the downstroke. As opposed to the above-mentioned studies, our measurements of deflection during the upstroke and downstroke were derived from free-flying insects and, therefore, the differences in deflection between the two half-strokes are not necessarily the result of forces with similar magnitudes.

Differences in force production between the upstroke and downstroke can result from changes in wing-tip velocity relative to air, as a result of the forward flight speed or due to changes in flapping kinematics (relative to the body) between the two half-strokes. The mean and maximal flight speed (±s.e., *n* = 14) of our beetles were 0.383 ± 0.031 m s^−1^ and 0.405 ± 0.033 m s^−1^, while the mean wing-tip velocity (see [72]) was 9.369 ± 0.178 m s^−1^, giving an advance ratio (mean forward flight speed/average wing-tip flapping speed; see [72]) of 0.041 ± 0.004. Ellington [72] proposed that flight at an advance ratio below a value of 0.1 can be considered hovering. Thus, forward (or backward) flight speed could only have had a minor effect on wing speed relative to air in our experiment. Similarly, the climb speed of the beetles had a negligible effect on the aerodynamic angle of attack and aerodynamic force production. This reflects on the fact that flight speed and acceleration during our take-off sequences were sufficiently low, so that in practice our take-off sequences were equivalent to hovering flight. Consequently, body mass, net vertical force (*F*_V_) and EAF yielded similar results as predictors of wing deformation, allowing to assess the scaling of wing deformation with body size. Inertial torques were found to scale with body mass approximately 1.0 and so did the aerodynamic forces. Hence, both the required aerodynamic force and the inertial torque increase in larger beetles requiring wing stiffening if the deformations of the larger wings are to remain proportional to wing length (span or chord).

The magnitude of chord-wise deflection at the trailing edge varied span-wise, with deflections increasing at the proximal sections of the wing. As the deflections are defined relative to the leading edge and distal sections of the wing are narrower, smaller chord-wise deflections at the wing tips are to be expected. However, in *P. cuprea* the large deflections at the proximal sections of the wing are clearly a result of reduced flexural stiffness in these areas, with fewer and thinner wing veins present in these sections (figure 1*a*). Directionality in span-wise flexural stiffness of the wings has been described before [68,71,73] for span-wise bending. It allows for twisting the wing about the median flexion line (see [26,68]), keeping the local angle of attack considerably constant despite span-wise differences in air speed associated with the flapping motion [39]. The most proximal section of the trailing edge has particularly low rigidity, resulting in delayed stroke reversal in this region (see film in electronic supplementary material, S8). It, therefore, resembles the pattern of wing rotation during clap and peal in lepidopteran flight [74,75] or perhaps the different rotation of the alula at the wing base of flies [76]. Insect wing rotation at the stroke reversal points is considered to be an important interaction of the wing surface with the vortical air structures it generates [49,77]. While the importance of wing rotation for insect flight is well known, how increased flexibility of proximal wing sections of the trailing edge affects the aerodynamics of stroke reversals is poorly understood.

**Figure 6.**
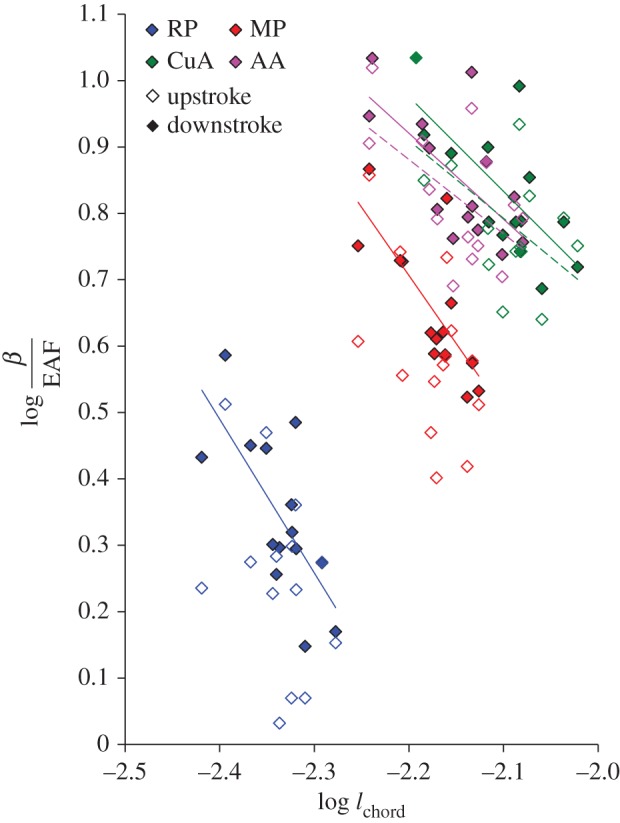
Logarithmic relationship between force-specific deflection (*β/*EAF) of landmarks and chord length (*l*_chord_). Full and open rhombi denote deflections during mid-downstroke and mid-upstroke, respectively. Colours and landmark names are as in figure 1*a*. Statistically significant linear regressions (on log-transformed data) are denoted by a trend line; see electronic supplementary material, S9 for elaborated statistics.

**Figure 7.**
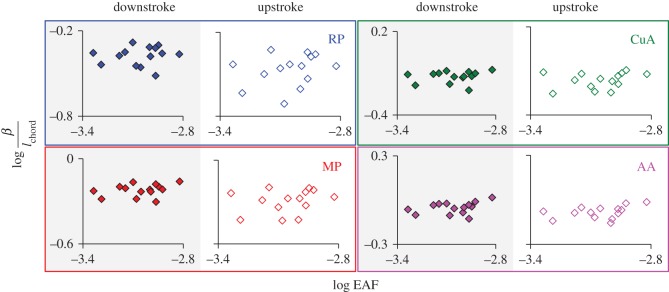
Logarithmic relationship between chord-length-specific deflection (*β/l*_chord_) at the trailing edge and the estimation of aerodynamic force (EAF). Colours and landmark names are as in figure 1*a*. None of the relationships were statistically significant; see electronic supplementary material, S10 for elaborated statistics.

Finally, our results demonstrate size-related changes in wing deflection. Not surprisingly, we found that the magnitude of deflection of the same homologous points on the trailing edge increased with body mass (and therefore wing size) and with the vertical force generated by the beetles. As explained in the Introduction, research on static bending of isolated insect wings and simplified scaling considerations suggest that chord-wise deflection may increase faster than the chord length. This would result in an increase in wing camber with beetle size. Using the data obtained from landmark CuA (figure 1*a*), we found that the deflection of the trailing edge at the proximal sections scaled with body mass ∼ 0.33 (the conclusion is similar when using *F*_V_ or EAF instead of body mass as the predictor; see electronic supplementary material, S6), implying a relationship with length ∼ 1. Thus, the increase in wing deflection with the size of *P. cuprea* was lower than our expectation. The increased rigidity of larger wings [23] was corroborated by our data showing a negative relationship between the force-specific deflection of the wing and chord length (figure 6). The outcome of this stiffening of the larger wings was a constant camber for the wings of beetles varying threefold in body mass (figure 7). Wing aspect ratio was independent of size (AR = 6.853 ± 0.038, Pearson correlation, *r* = −0.148, *p* = 0.572, *n* = 17), suggesting that span-wise and chord-wise growth in wing area remained isometric.

Because our measurements of wing deflection were performed during a climb, immediately after take-off, lift production of the beetle wings may be close to maximum [78]. Therefore, it is possible that maximal lift leads to a maximal wing deflection angle cos⁡(deflection/chord length), and the latter is a trait that is independent of body size. Nevertheless, the relationship between forces and deflections should not increase linearly if the flexural stiffness of the wing remains the same (see Introduction). Hence, stiffening of the wings of larger beetles is necessary for having similar maximum wing deflection angles.

We found no evidence for sexual dimorphism in the wing morphology of *P. cuprea* and therefore the data of males and females were pooled together to find the allometric relationships between wing deflection and body size. We found no significant effect of the beetles' sex on force-specific deflection (β/EAF) after controlling for the effect of chord length (*l*_chord_) in any of the four trailing edge landmarks (ANCOVA, *n*_(females)_ = 8, *n*_(males)_ = 4; RP: *F*_1,9_ = 2.224, *p* = 0.170; MP: *F*_1,9_ < 0.001, *p* = 0.994; CuA: *F*_1,9_ = 0.416, *p* = 0.535; AA: *F*_1,9_ = 0.278, *p* = 0.610). Similarly, chord-specific deflection $\left(\beta \text{/}l_{\mathrm{chord}}\right)$ was unaffected by the beetles' sex (Mann–Whitney *U* test: *p*-value > 0.68 for all four landmarks). Hence, although our sample size does not allow for a rigorous examination of sexual dimorphism in wing deflection in this species, it does not seem that the sex of the beetles used in our study could have biased our scaling relationships.

It seems plausible to assume that there is an optimal wing twist and camber for aerodynamic force productions in similarly shaped beetles and that the wings' flexural stiffness is adjusted to maintain these wing profile properties as the beetles increase in size. Lehmann *et al*. [73] found that the increase in local strain energy from the wing tip to the base matches the span-wise distribution of resilin along the wing. Resilin is a protein known to function as an elastic element in insect skeletons. Thus, it can adjust the local flexural stiffness of the wing, resulting in deviations from predictions based on uniform beams. Undoubtedly, insect wings are complex elastic structures; regardless, our results demonstrate that naturally occurring elastic wing deformations during free flight conserve the geometrical shape of the flapping wing as the insect grows in size. Our ongoing research seeks to reveal whether the size-invariable constant camber and twist pattern found here is unique to the Cetoniinae or is a general trend in insect flapping flight.

## Supplementary Material

Supporting materials 1-7

## Supplementary Material

Supporting materials 9-10

## Data accessibility

The data derived from this study are provided in the electronic supplementary material and in Dryad: (http://doi.org/10.5061/dryad.qk7g8) [79].

## Appendix A. Extracting wingbeat kinematics in the body frame of reference from the films

We used the three points on the thorax (figure 1*a*) to define a Cartesian coordinate system in which the longitudinal body axis (*x*) is represented by a vector connecting the scutellum with a point half-way between the two points on the pronotum. The lateral body axis (*y*) was defined as the vector connecting the right and left points on the pronotum. The dorsoventral body axis (*z*) was found from the cross product

A 1 z=x×y.

The origin was defined at the left wing base and all body axes vectors were converted to a unit vector. The vector cosines of the three body axes were used as a rotation matrix to convert each wing position to the body frame of reference (*x, y, z*). Thus, any position on the wing in the camera frame of reference *P*(*X, Y, Z*) is rotated to *P′*(*x, y, z*) in the body frame of reference according to

A 2 $$P^{\prime }=\left[\begin{array}{ccc} x_{X} & x_{Y} & x_{Z}\\ y_{X} & y_{Y} & y_{Z}\\ z_{X} & z_{Y} & z_{Z} \end{array}\right]\;\left[\begin{array}{c} P_{X}\\ P_{Y}\\ P_{Z} \end{array}\right].$$

The procedure is repeated for each frame, allowing extraction of the flapping kinematics of the wing in a frame of reference moving with the rigid body.

The instantaneous wing-tip data in the sagittal plane wt(x, z) were used to find the stroke plane from the linear least square error line of these positions throughout the three flapping cycles. The stroke-plane angle (SPA) is defined as the slope of the line as in Ellington [72], but relative to the body.

Next, we rotated wing data by SPA about the *y*-axis. The instantaneous flapping and deviation angles are defined as the azimuth and elevation of the wing length relative to the stroke plane. Wing pitch is defined as the rotation of the wing about its length. To find this angle in each film frame, we rotated the wing about the *z*-axis by the flapping angle so that the span aligned with the *y*-axis. We then found the vector connecting MP (figure 1*a*) with a point on the leading edge that is 0.73 of the distance from the wing base to the wing tip. The angle between this vector and the stroke plane in the *xz* plane is the instantaneous wing pitch.

## Appendix B. Deflection of points on the trailing edge relative to the leading edge

To measure chord-wise deflection of the wing, we first shifted the origin of all the position data to the wing base (point wb in figure 1*a*). We then defined two vectors connecting wb with two non-colinear points on the leading edge (mj and wt in figure 1*a*). The cross product of the two vectors defines a vector (*Z*_w_) normal to the plane formed by the three points (yellow triangle in figure 1*a*).

B 1 $$Z_{w}=\mathrm{mj}\times \mathrm{wt}.$$

The direction of the wing chord (*Y*_w_) is defined as the cross product of *Z*_w_ and wt:

B 2 $$Y_{w}=Z_{w}\times \mathrm{wt},$$

where all vectors are converted to unit vectors.

Using the vector cosines of the three wing axes (wt, *Y*_w_, *Z*_w_) as a rotation matrix, we transformed all data points on the wing to the wing frame of reference defined by the leading edge. The transformation of a given point P from the frame of reference of the cameras to the frame of reference of the leading edge (*P′*) is

B 3 $$P^{\prime }=\left[\begin{array}{ccc} wt_{X} & wt_{Y} & wt_{Z}\\ Yw_{X} & Yw_{Y} & Yw_{Z}\\ Zw_{X} & Zw_{Y} & Zw_{Z} \end{array}\right]\left[\begin{array}{c} P_{X}\\ P_{Y}\\ P_{Z} \end{array}\right].$$

The procedure is repeated for each film frame. By definition, the *Z*_w_ component of the transformed data points is the deflection of this point out of the plane of the rigid leading edge.
