# Supplementary material for: Allometry of wing twist and camber in a flower chafer during free flight: How do wing deformations scale with body size?
Source: R Soc Open Sci. 2017 Oct 18;4(10):171152. doi: 10.1098/rsos.171152 (PMC5666286; doi:10.1098/rsos.171152)
Supplement: Supporting materials 1-7 [file rsos171152supp1.docx]

**Supplementary materials for ”Allometry of wing twist and camber in a flower chafer during free-flight: How do wing deformations scale with body size?”**

**Supplementary material 1: Figure 2 of the main text re-drawn after calculating the plane of the rigid wing based on point RP instead of wt.** Colours and landmark names are as in figure 1 in the main text.


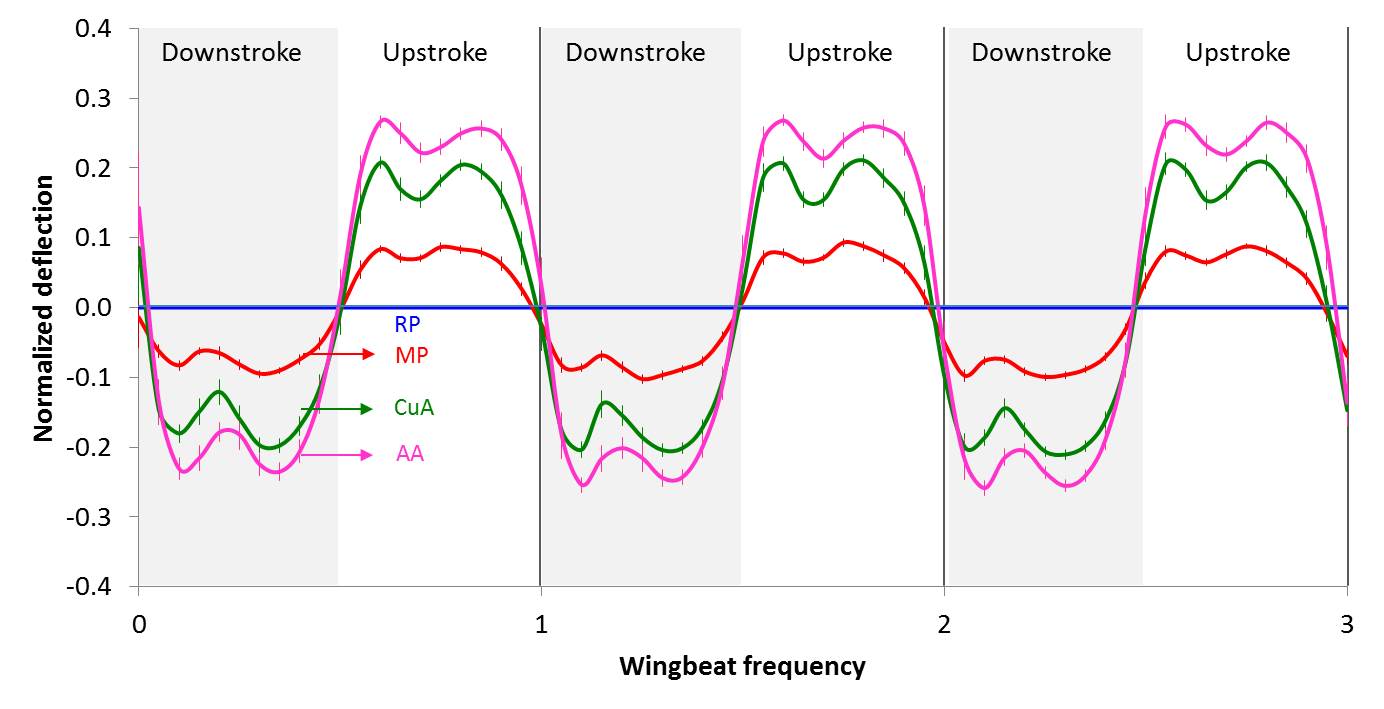


**Supplementary material 2: Normalized deflections of wing landmark from the rigid leading-edge plane.** The trailing-edge landmarks: RP, MP, CuA, and AA are marked with blue, red, green, and magenta, respectively. Three additional landmarks from the leading-edge between mj and wt are marked with dashed black lines. The weight of each beetle is shown in the top-left corner of each plot. The three plots denote the smallest, largest, and an intermediate beetle sizes. Colours and landmark names are as in figure 1 in the main text.

**
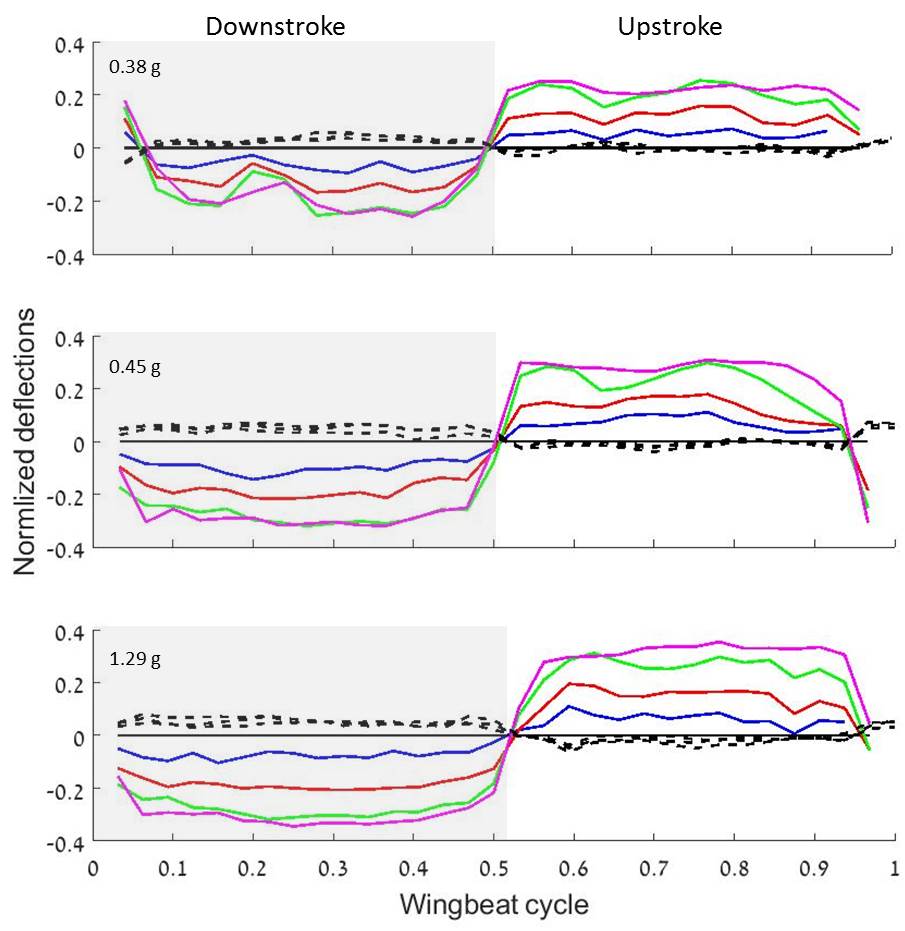
**

**Supplementary material 3: Lift and drag coefficients of insect wings as functions of wing's angle of attack.** For lack of precise data, the relationship between the lift and drag coefficients of the beetles’ wing and the angle of attack of the wing was generalized from figure 9 in (1) where data on these coefficients is summarized from various studies on flapping insect wings. We found that the general trigonometric relationships:

$C_{L}=3\sin\alpha\cos\alpha$ (S1a)

$C_{D}=3.35{sin}^{2}\alpha+0.1$ (S1b)

Provides a reasonable compromise between published fruit-fly (Reynolds number =~150) and the Hawkmoth (Reynolds number =~8000) data. The curves of Equations S1a and S1b are shown below in black (lift coefficient = open circles; drag coefficient = full circles). For comparison, grey symbols depict the curves for fruit-flies (2).

**
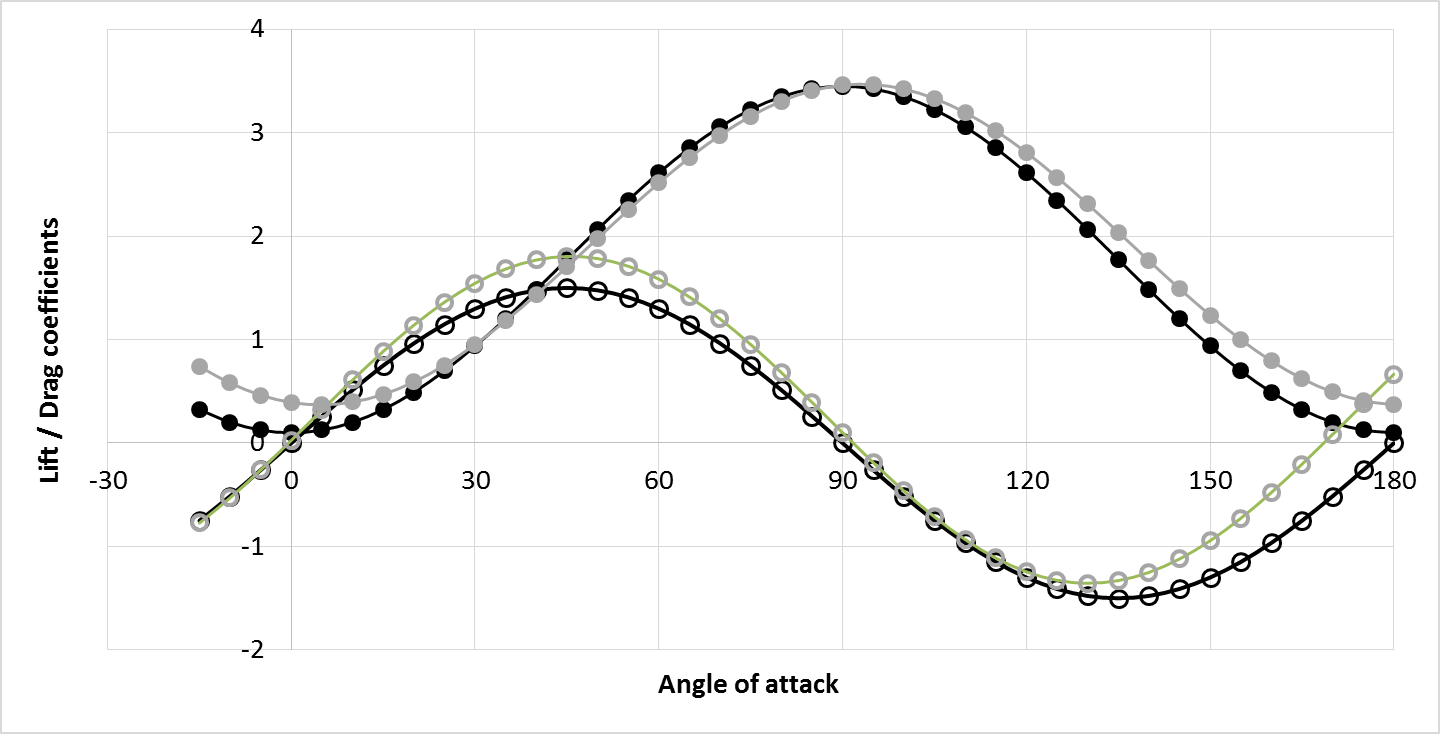
**

**Supplementary material 4: Calculation of wing inertial torques**

This section describes the measurements and calculations performed to find the mean inertial torque of the flapping wing and how it scales with beetle size. The inertial torque is important in bending the flexible wing (3). The elastic inertial bending of the wing along the chord and span are unlikely to be independent and although numerical and analytical solutions exist for simplified wings (3) we are unaware of a simple solution to estimate the inertial torque responsible for chord-wise deflection of an insect wing. Furthermore, these torques are generated at stroke reversals which are less than 20% of the flapping cycle duration and not during the mid-stroke where our deformation measurements have been performed. However, our results do show that chord-wise deflection varies along the span of the wings. Hence, we focus here on span-wise distribution of wing mass and the resulting span-wise bending torque during flapping. The increase in this bending torque with beetle size should hint at the contribution of inertial torques to the scaling of wing deformation. The technique followed is the one suggested by Ellington (4) with some minor modifications in the calculation of the distribution of wing mass.

**SM 4.1: Measurements**

Five beetles were killed in ethyl acetate vapors. Shortly after, we extracted the left wing and cut it into 4 parts at the landmark used for digitization (Fig. SM 4.1.1). We weighted the wing segments using an analytical balance (BOECO Germany, BBX 22, readability: 1x10^-5^ g). Then, we scanned the cut wing segments and measured them using an image analysis shareware (ImageJ, version 1.51k). The figure in SM 4.1.1 shows the four wing sections and measured distances and the tables in SM 4.1.2 present the measured data.

**SM 4.1.1: Markings illustrating cutting lines and measurements on the wings.**


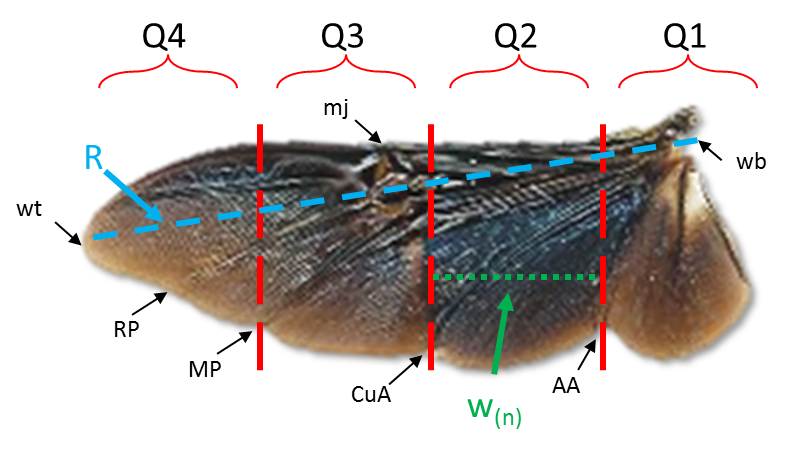


Dashed red lines mark the cutting lines that divide the wing length into four quarters (Q1-Q4). Black arrows point to the landmarks used in the study and correspond to those in figure 1 in the main text. Dashed blue line denotes wing length (R) and dotted green line w_(n)_ denotes the width of each wing section. $l_{(n)}$ is defined as the distance between the centroid of the n-th section and the wing base (not shown).

**SM 4.1.2: Mass and length measurements:** abbreviations correspond to SM 4.1.1

| Mass (kg) | | | | | | | | | | | | | | |
| --- | --- | --- | --- | --- | --- | --- | --- | --- | --- | --- | --- | --- | --- | --- |
| Body mass | Initial wing mass | | Q1 | | Q2 | | Q3 | | Q4 | | Sum of Q1-Q4 | | | % loss |
| 6.72E-04 | 4.13E-06 | | 1.38E-06 | | 1.10E-06 | | 8.10E-07 | | 2.60E-07 | | 3.90E-06 | | | 5.57 |
| 1.01E-03 | 5.41E-06 | | 2.64E-06 | | 1.55E-06 | | 8.10E-07 | | 1.00E-07 | | 5.01E-06 | | | 7.39 |
| 4.13E-04 | 1.82E-06 | | 6.70E-07 | | 5.00E-07 | | 3.30E-07 | | 0.00E+00 | | 1.60E-06 | | | 12.09 |
| 5.05E-04 | 2.54E-06 | | 9.90E-07 | | 1.17E-06 | | 5.70E-07 | | 1.80E-07 | | 2.42E-06 | | | 4.72 |
| 6.97E-04 | 2.97E-06 | | 1.31E-06 | | 8.80E-07 | | 4.90E-07 | | 0.00E+00 | | 2.78E-06 | | | 6.40 |
| Length (m) | | | | | | | | | | | | | | |
| Wing | $w_{Q1}$ | $w_{Q2}$ | | $w_{Q3}$ | | $w_{Q4}$ | | $l_{Q1}^{2}$ | | $l_{Q2}^{2}$ | | $l_{Q3}^{2}$ | $l_{Q4}^{2}$ | |
| 0.0229 | 0.0042 | 0.0059 | | 0.0059 | | 0.0070 | | 0.0021 | | 0.0072 | | 0.0131 | 0.0195 | |
| 0.0249 | 0.0053 | 0.0067 | | 0.0068 | | 0.0065 | | 0.0026 | | 0.0086 | | 0.0154 | 0.0221 | |
| 0.0180 | 0.0036 | 0.0043 | | 0.0046 | | 0.0053 | | 0.0018 | | 0.0058 | | 0.0102 | 0.0152 | |
| 0.0220 | 0.0047 | 0.0058 | | 0.0051 | | 0.0067 | | 0.0023 | | 0.0076 | | 0.0130 | 0.0189 | |
| 0.0219 | 0.0051 | 0.0054 | | 0.0059 | | 0.0060 | | 0.0025 | | 0.0078 | | 0.0134 | 0.0194 | |

**SM 4.2: Data analysis**

We calculated the wing’s mass moment of inertia ($I_{w}$), about the wing hinge, using the measurement of the four wing sections according to the parallel axis theorem

$$I_{w}=\sum_{n=1}^{n=4} \frac{1}{12}m_{n}\times{w_{n}}^{2}+m_{n}\times{l_{n}}^{2}$$

where $m_{n}$ is the mass (in kg) of each wing section, $w_{n}$is the width (in m) of each wing section and $l_{n}$ is the distance (in m) between the centroids of the n-th wing section and the wing base (wb). The sum of masses of the sections (after cutting the wing) was 5-12% smaller compared to the initial mass of the uncut wing (see Table in SM 4.1.2). Therefore, we corrected the masses of all wing sections by adding the % weight loss. Next, we calculated the non-dimensional radius of the 2^nd^ moment of inertia ($\hat{r}_{2}$) as in (Ellington 1984)(4,5):

$$\hat{r}_{2}\left( m \right)=\sqrt{\frac{I_{w}}{m_{w}\times R^{2}}}$$

Where $m_{w}$ is the wing mass (in kg) and *R* in wing length (in m; see SM 4.1.1). Wing thickness ($\hat{h}$) was calculated using the formula (Ellington 1984):

$$\hat{h}=\frac{m_{w}\times AR}{4\times\rho_{w}\times R^{3}}$$

where *AR* is the aspect ratio, $\rho_{w}$ is wing density (=1200 kg m^-3^ for insect cuticle; see review by Ellington (4,5).

Taking the mean of the last two variables ($\hat{r}_{2}$ & $\hat{h}$) of the 5 beetle measured we calculated the mass moment of wing inertia (*I_w_*) for each of the 12 beetles in the films according to Ellington (4,5) as:

$$I_{w}=m_{2}=\rho_{w}\times S\times R^{3}\times\hat{h}\times\left( \hat{r}_{2}\left( m \right) \right)^{2}$$

where $S$ is the wing area of each beetle in the movies and the non-dimensional expression $\hat{h}\times\left( \hat{r}_{2}\left( m \right) \right)^{2}$ was taken from the mean of the 5 beetle wings measured for wing mass distribution ($1.56\times{10}^{-4}$ ). Consequently, the inertial torque ($\tau_{I}$) for each of the filmed beetles was calculated as in (4,5):

$$\tau_{I}=\frac{\frac{1}{2}I_{w}\left( 2\times\Omega\times f \right)^{2}}{\frac{\Omega}{2}}=4{\times I}_{w}\times\Omega{\times f}^{2}$$

where $\Omega$ is the flapping amplitude (in radians) and $f$ is wing-beat frequency (in Hz).

We did not include virtual mass in the calculation because this added mass for a cambered wing was expected to be small and depend on the assumed shape of the deflected wing.

Note that $\tau_{I}$ is dimensionally the same as the simpler result given by Daniel and Combes (3) for the oscillatory moment at the wing base required to flap a rectangular plate.

The use of non-dimensional parameters above and the allometric equations (equations 7 & 8) found in the main text shows that:

$$\tau_{I}\propto R^{5}{\times f}^{2} {\to\tau}_{I}\propto{{(M}^{0.246})}^{5}{{(M}^{-0.137})}^{2}$$

and therefore $\tau_{I}\propto M^{0.96}$; I.e. the inertial torque of the wings (which is independent of the aerodynamic force) should increase linearly with body mass (or length^3^). Figure SM 4.2.1 shows the actual relationship using the flapping kinematics of our beetles.

Combes and Daniel (6) found that the flexural stiffness of insect wings scales with the cubic power of wing span and square power of wing chord. For a cantilever beam the relationship between deflection and point load (equation 1 in the main text) can be rewritten as:

$$\beta=\frac{{\tau_{I}l}^{2}}{8EI}$$

If the inertial torque is proportional to length^3^, and *EI* to length^2.0-3.0^ the deflection ($\beta)$ should increase faster than wing span or chord. The fact that our results do not portray this increase supports our conclusion that the wings of larger insects are stiffer than expected.

The above calculation of wing inertial torque is based on span-wise bending due to the angular acceleration of the wing during the quarter strokes. At stroke reversals the angular acceleration is maximal and during the half stroke the instantaneous inertial torque should be negligible.

**SM 4.2.1: Relationships between inertial torque vs. body mass and inertial torque vs. vertical force (*F_V_*)**.


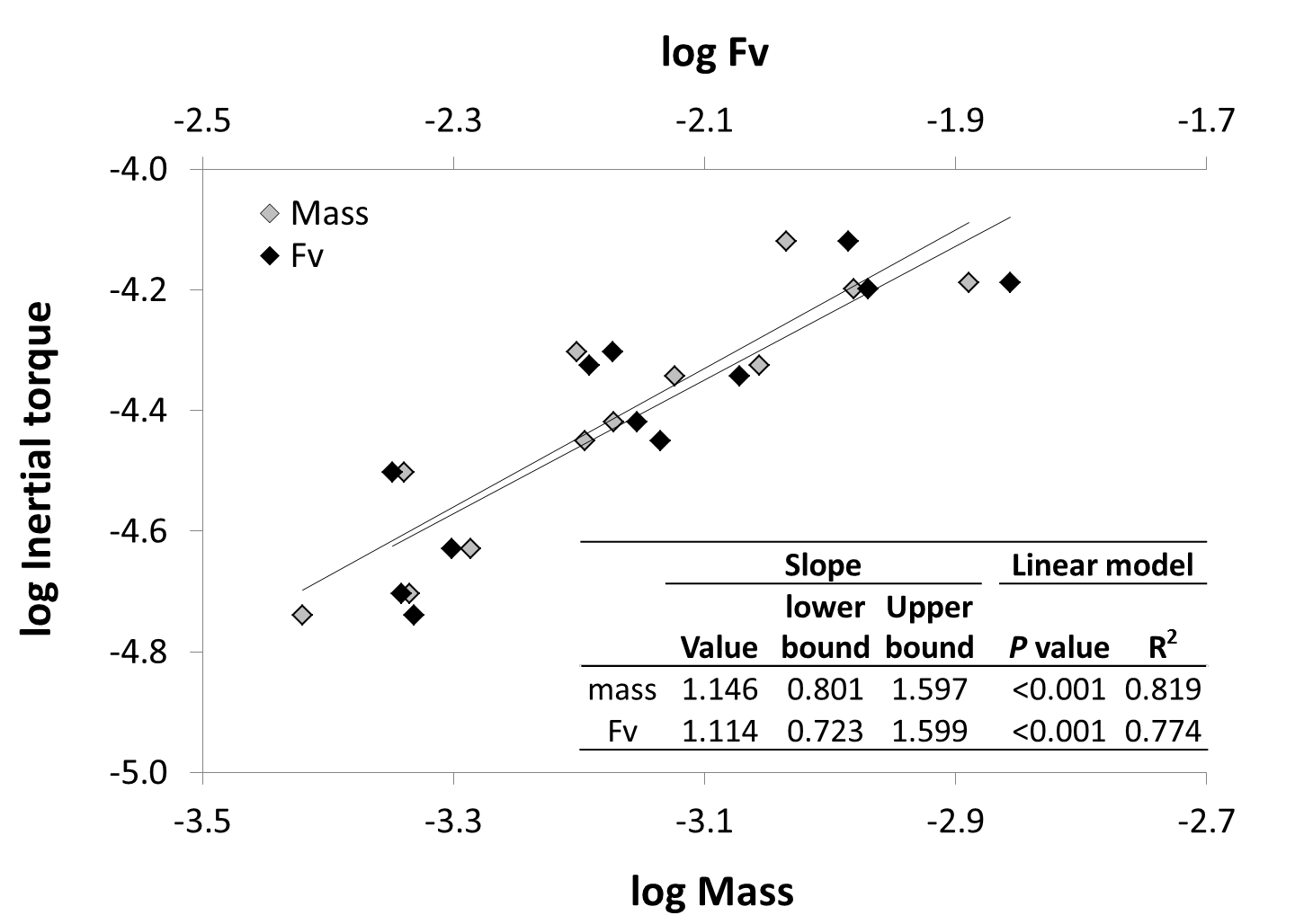


Linear regressions were performed on log-transformed data. Legend and statistics are embedded in the figure.

**SM 4.3: Data and results for inertial torque calculation**

| Body mass (kg) | Wing length (m) | Wing area  (m^2^) | Aspect ratio | Moment of inertia | Amplitude (radians) | Frequency (Hz) | Inertial Force |
| --- | --- | --- | --- | --- | --- | --- | --- |
| 8.79E-04 | 2.54E-02 | 1.88E-04 | 6.873 | 5.78E-10 | 1.954 | 102 | 4.72E-05 |
| 3.80E-04 | 1.95E-02 | 1.10E-04 | 6.898 | 1.52E-10 | 2.022 | 122 | 1.82E-05 |
| 6.72E-04 | 2.40E-02 | 1.65E-04 | 7.004 | 4.29E-10 | 1.842 | 110 | 3.81E-05 |
| 4.58E-04 | 2.30E-02 | 1.51E-04 | 6.976 | 3.44E-10 | 1.994 | 107 | 3.15E-05 |
| 6.38E-04 | 2.34E-02 | 1.57E-04 | 6.986 | 3.76E-10 | 1.956 | 110 | 3.55E-05 |
| 4.63E-04 | 1.96E-02 | 1.13E-04 | 6.759 | 1.60E-10 | 1.871 | 129 | 1.98E-05 |
| 6.28E-04 | 2.50E-02 | 1.78E-04 | 7.025 | 5.20E-10 | 1.941 | 111 | 4.98E-05 |
| 1.29E-03 | 2.70E-02 | 2.13E-04 | 6.825 | 7.88E-10 | 2.103 | 99 | 6.48E-05 |
| 9.24E-04 | 2.74E-02 | 2.23E-04 | 6.744 | 8.61E-10 | 1.742 | 113 | 7.60E-05 |
| 5.18E-04 | 2.20E-02 | 1.38E-04 | 6.998 | 2.77E-10 | 2.074 | 101 | 2.35E-05 |
| 1.04E-03 | 2.68E-02 | 2.17E-04 | 6.599 | 7.83E-10 | 2.066 | 99 | 6.33E-05 |
| 7.53E-04 | 2.43E-02 | 1.75E-04 | 6.780 | 4.73E-10 | 2.139 | 106 | 4.54E-05 |

**Supplementary material 5: Difference in trailing-edge deflections along wing span.**

Paired comparisons (two-tailed t-tests) of the deflection of landmarks (distal minus proximal) along the trailing edge. Data for mid-downstroke and mid-upstroke are compared separately. Bonferroni correction due to multiple comparisons sets the significance level (alpha) to 0.0083.

|  |  | Paired differences (m) | |  |  |
| --- | --- | --- | --- | --- | --- |
| Stroke | Landmarks | Mean | s.e. | *t* value | *p* value |
| Downstroke | RP – MP | -2.1E-03 | 9.9E-05 | -20.835 | <0.001 |
|  | RP – CuA | -4.1E-03 | 1.8E-04 | -22.193 | <0.001 |
|  | RP – AA | -4.4E-03 | 2.3E-04 | -18.796 | <0.001 |
|  | MP – CuA | -2.0E-03 | 9.9E-05 | -20.127 | <0.001 |
|  | MP – AA | -2.3E-03 | 1.5E-04 | -15.621 | <0.001 |
|  | CuA – AA | -3.4E-04 | 7.5E-05 | -4.461 | 0.001 |
| Upstroke | RP – MP | -1.8E-03 | 9.7E-05 | -18.914 | <0.001 |
|  | RP – CuA | -3.9E-03 | 1.9E-04 | -21.055 | <0.001 |
|  | RP – AA | -4.3E-03 | 2.2E-04 | -20.089 | <0.001 |
|  | MP – CuA | -2.1E-03 | 1.1E-04 | -18.402 | <0.001 |
|  | MP – AA | -2.5E-03 | 1.7E-04 | -15.002 | <0.001 |
|  | CuA – AA | -4.2E-04 | 9.3E-05 | -4.532 | 0.001 |

**Supplementary material 6: Body mass, vertical force (*F_V_*), and estimation of aerodynamic force (*EAF*) as predictors of deflection of the trailing edge during the downstroke.** Colours and landmark names are the same as in figure 1*a* (see main text). Regression lines are displayed if statistically significant (*P*<0.05). The table below summarises the statistics. All linear models are based on 1000 bootstrap samples.

**
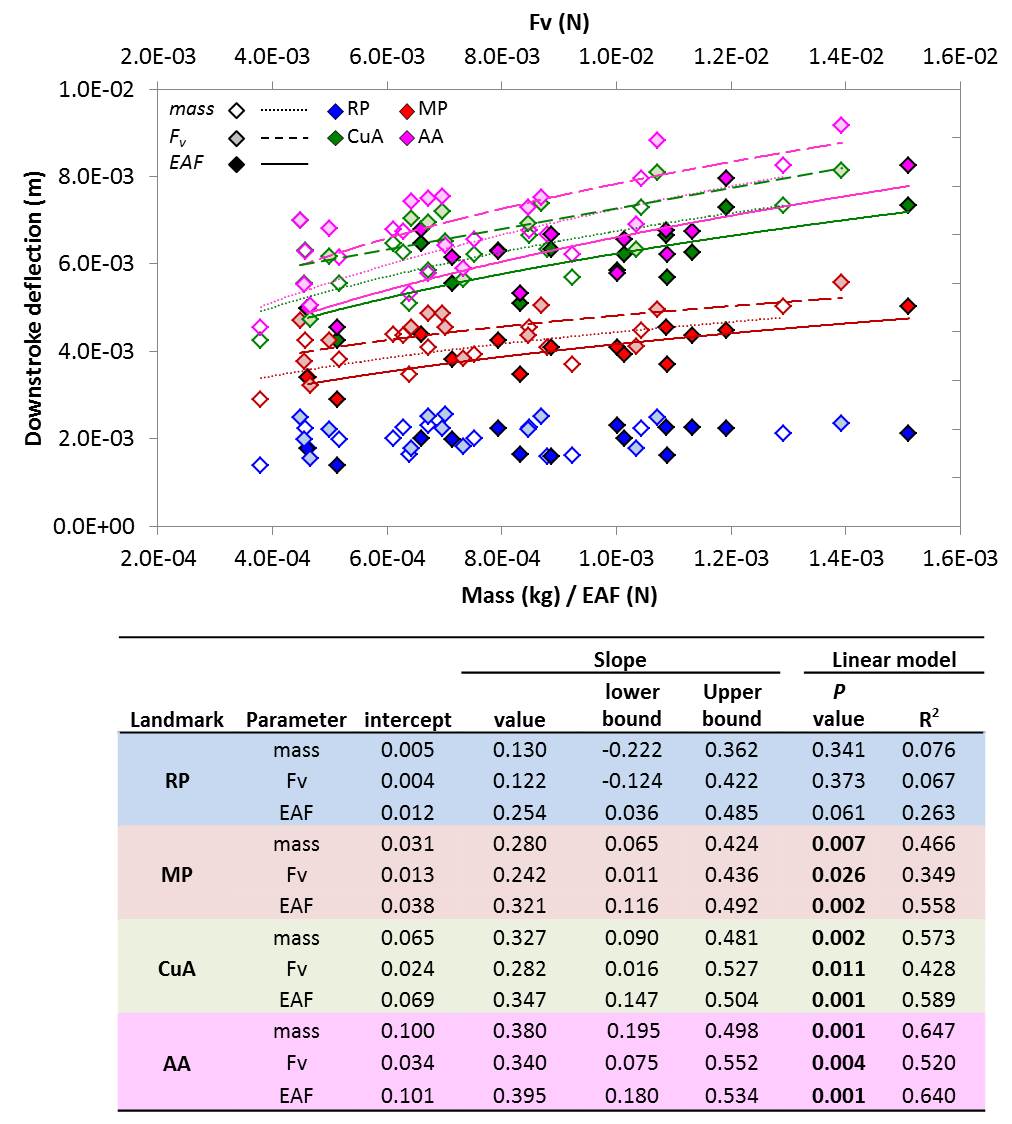
**

**Supplementary material 7: Body mass and vertical force (*F_V_*) as predictors of estimated aerodynamic force (*EAF*).** *EAF* is calculated from equation 2 and *F_V_* from the body mass and acceleration of the body (see main text) Grey and black rhombi, and dashed and solid regression lines denote the *EAF*-mass and *EAF*- *F_V_* relationships, respectively.


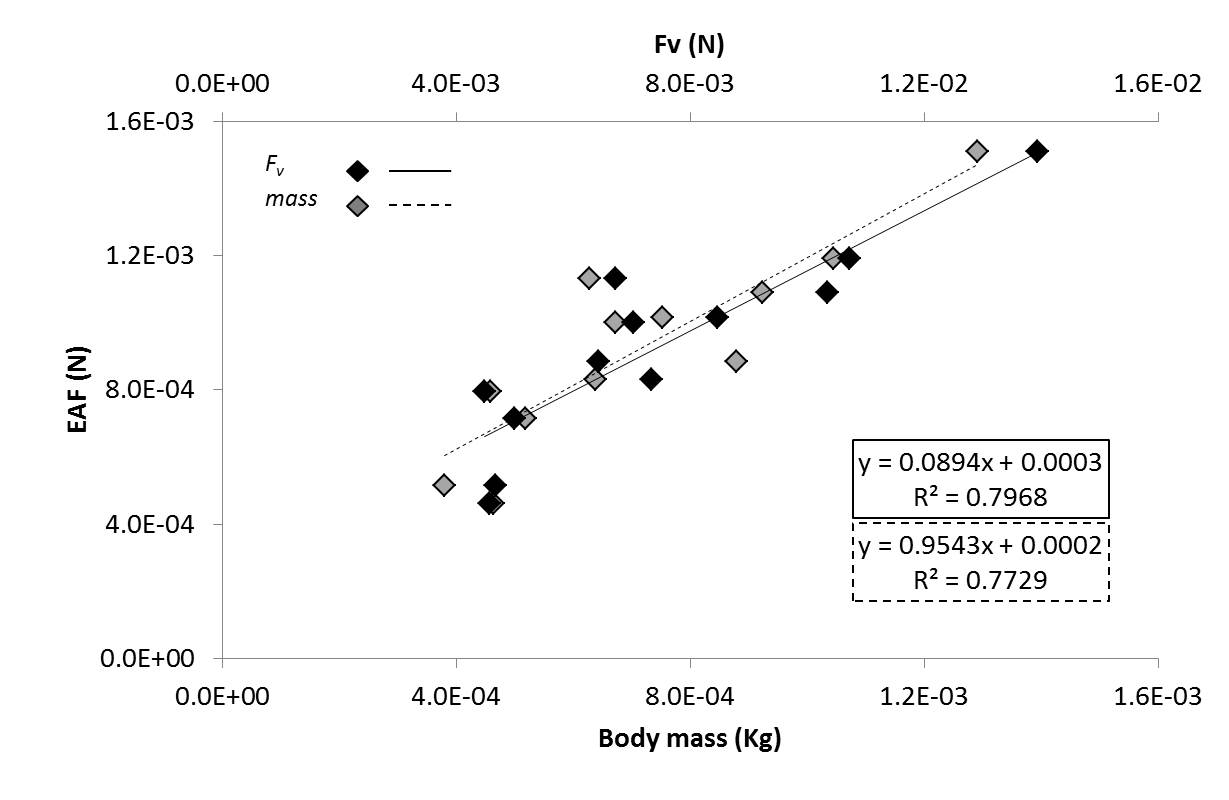


**References**

1. Sane SP. 2003 The aerodynamics of insect flight. *J Exp Biol*, **206**, 4191–208. (doi:10.1242/jeb.00663(
2. Dickinson MH, Lehmann FO, Sane SP. 1999 Wing Rotation and the Aerodynamic Basis of Insect Flight. *Science*. **284**, 1954–60. (doi:10.1126/science.284.5422.1954)
3. Daniel TL, Combes SA. 2002 Flexible wings and fins: bending by inertial or fluid-dynamic forces? *Integr Comp Biol*. **42**, 1044–9. (doi:10.1093/icb/42.5.1044).
4. Ellington C. 1984 The aerodynamics of hovering insect flight. II. Morphological parameters. *Philos Trans R Soc B Biol Sci.* **305**, 17–40.
5. Ellington C. 1984 The aerodynamics of hovering insect flight. VI. Lift and power requirements. *Philos Trans R Soc B Biol Sci*. **305**, 145–81.
6. Combes SA, Daniel TL. 2003 Flexural stiffness in insect wings I. Scaling and the influence of wing venation. *J Exp Biol.* **206**, 2979–87. (doi:10.1242/jeb.00523).
