# Supplementary material for: Allometry of wing twist and camber in a flower chafer during free flight: How do wing deformations scale with body size?
Source: R Soc Open Sci. 2017 Oct 18;4(10):171152. doi: 10.1098/rsos.171152 (PMC5666286; doi:10.1098/rsos.171152)
Supplement: Supporting materials 9-10 [file rsos171152supp2.docx]

**Supplementary materials for ”Allometry of wing twist and camber in a flower chafer during free-flight: How do wing deformations scale with body size?”**

**Supplementary material 9: Elaborated statistics for Figure 6 in the main text.** The linearity tests are between the force specific deflections ($\frac{\beta}{F_{EAF}}$) and chord length ($l_{chord}$) after log transforming the data. Replacing *EAF* with net vertical fore (*F_v_*) or body mass gave similar results. Statistical significance (*p* < 0.05) is highlighted in bold. All the results are based on 1000 bootstrap samples.

| Landmark | Stroke | Slope | lower bound | Upper bound | R^2^ | *p* value |
| --- | --- | --- | --- | --- | --- | --- |
| RP | Down | -2.288 | -3.822 | -1.025 | 0.487 | **0.006** |
|  | Up | -1.426 | -3.778 | 0.139 | 0.145 | 0.179 |
| MP | Down | -2.100 | -3.004 | -1.260 | 0.566 | **0.002** |
|  | Up | -1.660 | -3.291 | 0.001 | 0.255 | 0.066 |
| CuA | Down | -1.421 | -2.038 | -0.652 | 0.482 | **0.006** |
|  | Up | -1.150 | -2.143 | 0.053 | 0.290 | **0.047** |
| AA | Down | -1.290 | -1.800 | -0.576 | 0.448 | **0.009** |
|  | Up | -1.138 | -1.882 | -0.131 | 0.327 | **0.033** |

**Supplementary material 10: Elaborated statistics for Figure 7 in the main text.**

The linearity tests are between the length specific deflections ($\frac{\beta}{l_{chord}}$) and estimated aerodynamic force ($EAF$) after log transforming the data. Replacing *EAF* with net vertical fore (*F_v_*) or body mass gave similar results. None of the relationships are statistically significant at the 95% confidence level. All the results are based on 1000 bootstrap samples.

| Landmark | Stroke | Slope | Lower bound | Upper bound | R^2^ | *p* value |
| --- | --- | --- | --- | --- | --- | --- |
| RP | Down | 0.057 | -0.173 | 0.270 | 0.014 | 0.683 |
|  | Up | 0.212 | -0.144 | 0.633 | 0.070 | 0.361 |
| MP | Down | 0.088 | -0.086 | 0.220 | 0.086 | 0.308 |
|  | Up | 0.145 | -0.140 | 0.509 | 0.060 | 0.397 |
| CuA | Down | 0.056 | -0.150 | 0.208 | 0.036 | 0.516 |
|  | Up | 0.096 | -0.142 | 0.362 | 0.063 | 0.386 |
| AA | Down | 0.095 | -0.108 | 0.231 | 0.115 | 0.235 |
|  | Up | 0.113 | -0.067 | 0.268 | 0.149 | 0.173 |
